# PD-0332991, a CDK4/6 Inhibitor, Significantly Prolongs Survival in a Genetically Engineered Mouse Model of Brainstem Glioma

**DOI:** 10.1371/journal.pone.0077639

**Published:** 2013-10-02

**Authors:** Kelly L. Barton, Katherine Misuraca, Francisco Cordero, Elena Dobrikova, Hooney D. Min, Matthias Gromeier, David G. Kirsch, Oren J. Becher

**Affiliations:** 1 Department of Pediatrics, Duke University, Durham, North Carolina, United States of America; 2 Department of Pathology, Duke University, Durham, North Carolina, United States of America; 3 Preston Robert Tisch Brain Tumor Center, Duke University, Durham, North Carolina, United States of America; 4 Graduate Program in Molecular Cancer Biology, Duke University, Durham, North Carolina, United States of America; 5 Department of Surgery, Duke University, Durham, North Carolina, United States of America; 6 Department of Radiation Oncology, Duke University, Durham, North Carolina, United States of America; 7 Department of Pharmacology and Cancer Biology, Duke University, Durham, North Carolina, United States of America; University Hospital of Navarra, Spain

## Abstract

Diffuse intrinsic pontine glioma (DIPG) is an incurable tumor that arises in the brainstem of children. To date there is not a single approved drug to effectively treat these tumors and thus novel therapies are desperately needed. Recent studies suggest that a significant fraction of these tumors contain alterations in cell cycle regulatory genes including amplification of the D-type cyclins and CDK4/6, and less commonly, loss of Ink4a-ARF leading to aberrant cell proliferation. In this study, we evaluated the therapeutic approach of targeting the cyclin-CDK-Retinoblastoma (Rb) pathway in a genetically engineered PDGF-B-driven brainstem glioma (BSG) mouse model. We found that PD-0332991 (PD), a CDK4/6 inhibitor, induces cell-cycle arrest in our PDGF-B; Ink4a-ARF deficient model both *in vitro* and *in vivo*. By contrast, the PDGF-B; p53 deficient model was mostly resistant to treatment with PD. We noted that a 7-day treatment course with PD significantly prolonged survival by 12% in the PDGF-B; Ink4a-ARF deficient BSG model. Furthermore, a single dose of 10 Gy radiation therapy (RT) followed by 7 days of treatment with PD increased the survival by 19% in comparison to RT alone. These findings provide the rationale for evaluating PD in children with Ink4a-ARF deficient gliomas.

## Introduction

High-grade brainstem gliomas (BSGs), also called diffuse intrinsic pontine glioma or DIPG when localized in the pons, account for up to 15-20% of pediatric brain tumors [1]. They are one of the leading causes of death among children with brain tumors with not a single drug approved [2]. Therefore, effective therapies are desperately needed for this disease.

Aberrant cell cycle control is a hallmark of human cancers. Cyclin dependent kinases (CDK) are essential for regulation of the cell cycle. Together D-type cyclins and CDK4/6 form complexes crucial for the G1-S phase transition. These complexes phosphorylate Rb, a product of the retinoblastoma protein tumor suppressor gene. In its active form, hypophosphorylated pRb is bound to E2Fs inhibiting their transcriptional activity. Hyperphosphorylation of pRb renders it inactive and allows for the release of E2F transcription factors necessary for initiation of S-phase and cell cycle progression [3]. An endogenous CDK4/6 inhibitor, p16^INK4A^, negatively regulates these processes. In recent years there have been numerous clinical trials in adults with cancer utilizing small molecule inhibitors targeting cell cycle regulatory genes such as CDKs. However, CDK inhibitors have yet to be evaluated in children with DIPG.

Recent studies of DIPG autopsy and biopsy tissues have unraveled the genetic alterations of DIPG [4-7]. These studies noted that approximately 30% of these tumors contain alterations in cell cycle regulatory genes including amplification of D-type cyclins and CDK4/6, or loss of Ink4a-ARF. Amplification of these proteins, or loss of Ink4a, results in abnormal proliferation. While Ink4a-ARF is rarely deleted at the genomic level in DIPG, a recent study has shown that Ink4a protein expression is lost through other mechanisms [6,8]. Most importantly, the above-mentioned studies noted that Rb1 deletions are rare suggesting that inhibition of the cyclin/CDK/Rb axis may be a viable option for the majority of DIPGs.

Therefore we were interested to evaluate the efficacy of a cyclin/CDK/Rb axis inhibitor in a genetically engineered mouse model of BSG, which is driven by PDGF-B overexpression and Ink4a-ARF loss [9]. As 77% of BSGs harbor p53 mutations [10], we were also interested in determining the efficacy in a PDGF-B; p53 deficient BSG model. To determine if targeting this pathway is a good approach to treating DIPG we selected the CDK4/6 inhibitor PD-0332991 (PD). PD is a highly selective, orally administered inhibitor of both CDK4 and CDK6 kinase activity and is currently being evaluated in clinical trials for a variety of adult cancers [11]. PD is selective for CDK4 and CDK6, with IC50 values for CDK4/cyclinD1, CDK4/cyclinD3, and CDK6/cyclinD2 of 11, 9, and 15 nM, respectively, and has low activity against a panel of 36 additional protein kinases, including CDK2/cyclin E2, CDK2/cyclin A, and CDK1/cyclin B [12]. In addition, recent publications suggest that PD has a good safety profile and demonstrates efficacy in cancers such as mantle cell lymphoma that harbor aberrant expression of cyclin D1 due to a translocation [13].

Since radiation therapy (RT), the current standard of care for children with DIPG provides only temporary relief, we were interested to evaluate whether treatment with PD immediately following RT would enhance the survival benefit of RT. Although there have been numerous clinical trials for DIPG combining chemotherapy or targeted agents with RT, none have successfully provided a survival advantage over radiation alone [1]. Here we evaluated the efficacy of PD alone and PD with RT in our immunocompetent, genetically relevant BSG mouse model.

## Results

In this study we used two distinct genetically engineered mouse models of BSGs generated with the RCAS/tv-a mouse modeling system. PDGF-B overexpression in nestin expressing cells of the neonatal brainstem, along with Ink4a-ARF deletion, leads to highly aggressive BSGs [9]. As a significant fraction of BSGs harbor p53 mutations we also generated a PDGF-B driven; p53 deficient BSG mouse model. The experiments conducted in this study were performed on tumor bearing mice or cell lines derived from tumor bearing mice of both models.

### CDK 4/6 and D-type cyclins are overexpressed in both PDGF-B-driven BSG mouse models

**Figure 1:**
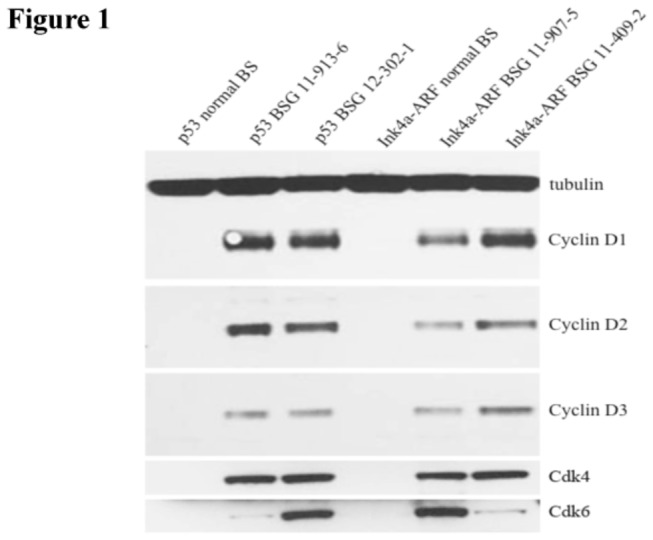
PDGF-B; Ink4a-ARF deficient and PDGF-B; p53 deficient BSG models overexpress all three D-type cyclins. Western blot for cyclins D1, D2, D3, CDK 4 and CDK 6. From left to right: lane 1 is normal brainstem from Ntv-a; p53 floxed mice, lanes 2 and 3 are two PDGF-B; p53 deficient BSGs, lane 4 is normal brainstem from Ntv-a; Ink4a-ARF deficient mice, lanes 5 and 6 are two PDGF-B; Ink4-ARF deficient BSGs.

We were first interested to determine if CDK4/6 and D-type cyclins are overexpressed at the protein level in two PDGF-B driven BSG mouse models: Ink4a-ARF deficient and p53 deficient. Interestingly, western blot analysis demonstrated that CDK4/6 and all three D-type cyclins were overexpressed in both BSG models relative to the normal brainstem (Figure 1). These results provided an initial rationale that a CDK4/6 inhibitor could be a potential therapeutic agent in the two BSG mouse models.

### Ink4a-ARF deficient BSG cell lines are more sensitive to PD than p53 deficient BSG cell lines

**Figure 2:**
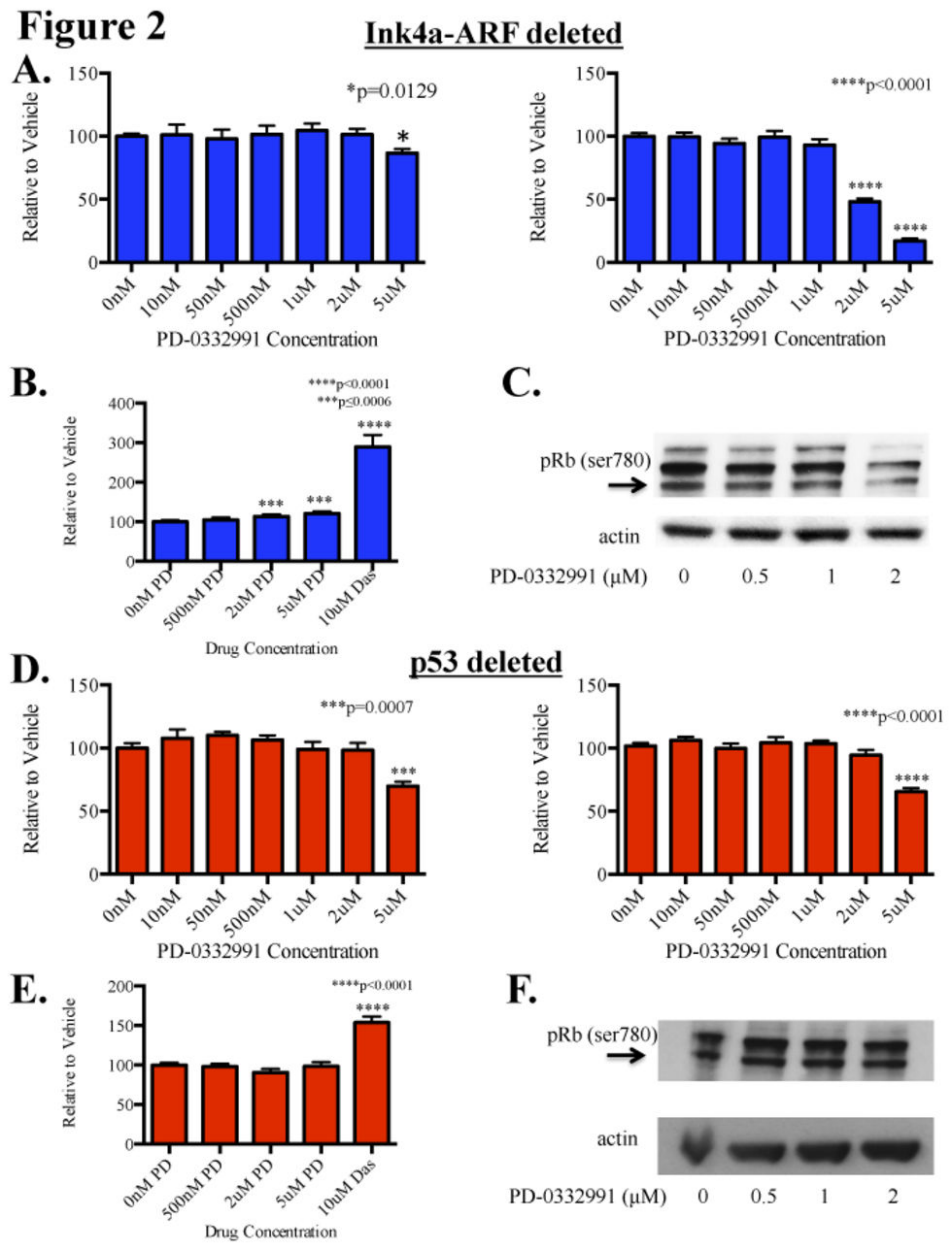
Effects of PD-0332991 on BSG cell lines derived from genetically engineered mouse models. Cells were treated with PD-0332991 for 48 hours before being harvested for MTT or BrdU assays to assess cell survival and proliferation, respectively. (A) MTT assays (left panel) show minimal cytotoxic activity at doses up to 5µM in cell lines derived from PDGF-B; Ink4a-ARF deficient BSGs. BrdU assays (right panel) showed inhibition of proliferation with an IC50 of 1.8µM. Assays performed in two independent cell lines. (B) Apoptosis assays showed a very small but significant increase in apoptosis at 2µM and 5µM (p=0.0006 and p=0.0002, respectively). (C) Western blot analysis shows inhibition of Rb phosphorylation at the protein level, with inhibition observed at 2µM in the PDGF-B; Ink4a-ARF deficient line after only 24 hours of treatment. (D) MTT assays (left panel) show the minimal cytotoxic activity only at doses of 5µM in cell lines derived from PDGF-B; p53 deficient BSGs. BrdU assays (right panel) showed significant inhibition of proliferation at 5µM but IC50 was not reached. (E) Apoptosis assays did not show any difference in apoptosis between vehicle and up to 5µM PD. Assays performed in two independent cell lines. Error bars represent SEM from three independent experiments. Statistical significance was determined using One-way ANOVA; paired student’s t-test was used to compare within groups. (F) PDGF-B; p53 deficient BSG cells show no decrease in pRb at doses up to 2µM even after 48 hours of treatment with PD.

Between successful early phase clinical trials with PD in adults, relevant murine models, and potential for therapeutic targeting in BSGs, we were interested to determine how PD would affect our BSG cell lines *in vitro*. To examine the efficacy of PD we performed a variety of *in vitro* assays on two independent PDGF-B driven Ink4a-ARF^-/-^ BSG cell lines. Using MTT assays, we observed that treatment with PD for 48 hours was minimally cytotoxic to the Ink4a-ARF deficient BSG cells at a dose of 5µM but not at lower doses (Figure 2A, left panel). We also noted that proliferation of these cells was indeed inhibited by PD, with an IC50 of 1.8µM as assessed with BrdU assays (Figure 2A, right panel). Since proliferation was inhibited, we were also interested to determine if PD induced apoptosis. Surprisingly, PD induced a very small but significant increase in caspase 3/7 activity levels at doses of 2µM and 5µM PD but not at 500nM (Figure 2B).

To assess if the mechanism of action of PD was in fact through inhibition of CDK 4 and 6 we treated cells with PD at increasing concentrations for 24 hours and looked at pRb levels at serine 780 (one of the phosphorylation sites of CDK 4 and 6). Consistent with the BrdU assays, we observed inhibition of pRb at 2µM (Figure 2C).

As 77% of BSGs harbor p53 mutations, we were also interested in determining the efficacy of PD in our PDGF-B; p53 deficient BSG cell lines. We again used two independent cell lines and repeated the same course of experiments. Similar to our observations with the PDGF-B; Ink4a-ARF deficient BSG cell lines, PD was only minimally cytotoxic to the PDGF-B; p53 deficient BSG cells at a dose of 5µM but not at lower doses (Figure 2D, left panel). In contrast, BrdU assays showed that p53 deficient cell lines were less sensitive to PD treatment; the IC50 was not achieved with doses up to 5µM (Figure 2D, right panel). Concordantly, we found that apoptosis was not induced in PDGF-B; p53 deficient BSG cells by treatment with PD (Figure 2E). To validate these findings we then examined pRb levels. After 48 hours of treatment, pRb inhibition was not achieved at doses up to 2µM (Figure 2F). Collectively, our results suggest that treatment with PD is more effective against PDGF-B; Ink4-ARF deficient cell lines than PDGF-B; p53 deficient cell lines.

### PD induces cell-cycle arrest in the Ink4a-ARF deficient BSG model

**Figure 3:**
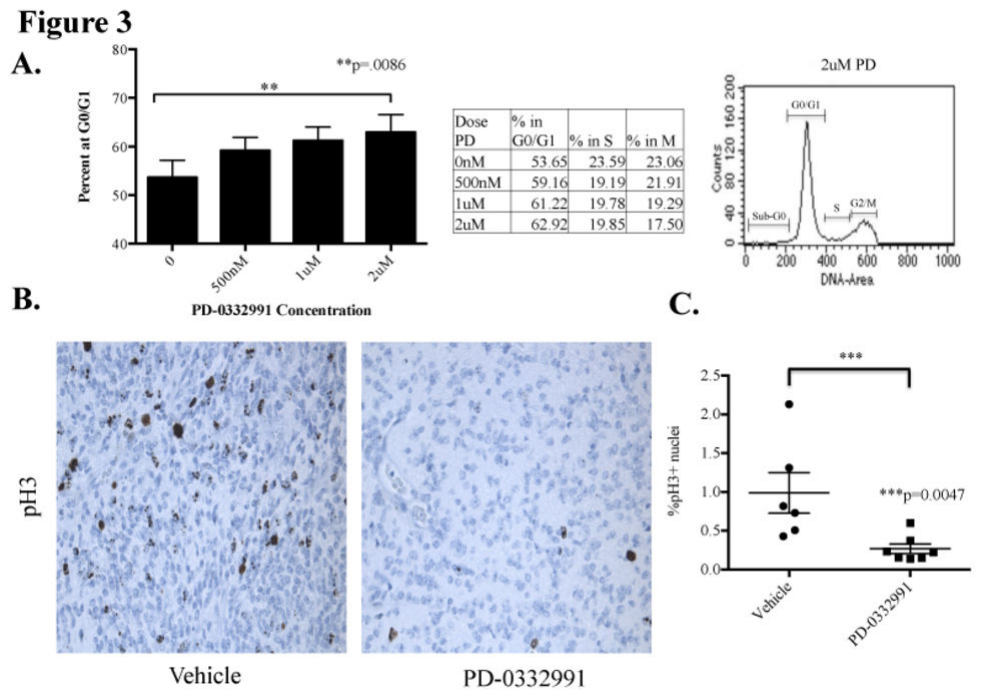
PD-0332991 induces a cell cycle arrest in the PDGF-B driven Ink4a-ARF deficient BSG model. (A) Cell cycle analysis of two independent Ink4a-ARF deficient BSG cell lines shows a significant increase in the percentage of cells at G0/G1, indicating cell cycle arrest, after 48 hours of treatment with PD-0332991. Table in the middle panel shows the mean of three independent experiments with two cell lines. A cell cycle analysis tracing (right panel) shows minimal apoptotic activity at 2µM. (B & C) Tumor bearing mice were given two treatments of PD-0332991 or vehicle, once daily by oral gavage (150mg/kg), and sacrificed 24 hours later. Brains were formalin fixed and paraffin embedded. (B) Representative phospho-histone 3 (pH3) staining, a marker for M phase, shows higher levels of proliferating cells in vehicle treated mice (left panel) as opposed to PD treated mice (right panel). (C) Quantification of immunostaining comparing the % pH3 positive nuclei/total nuclear area using MetaMorph Image Analysis Software. Statistics were done using a Mann-Whitney test and horizontal bars represent mean with SEM.

Upon observing that PDGF-B; Ink4a-ARF deficient BSG cells were more sensitive to PD, we continued our study with this model. To confirm our observations with the BrdU assay, we performed cell cycle analysis. Indeed, cell cycle analysis of PDGF-B; Ink4a-ARF deficient cell lines treated with PD demonstrated a significant increase in the percentage of cells in G0/G1 in response to a 48-hour treatment with 2µM PD (Figure 3A, left panel). We also noted that while it was not significant, there was a trend of a decrease in the percentage of cells in M phase (Figure 3A, middle panel). Lastly, we did not observe a sub-G0 population in the cell cycle analysis at PD doses up to 2µM.

While the *in vitro* efficacy was encouraging, ultimately drug delivery into the brainstem is thought to be the major barrier for progress against DIPGs. Therefore, we were interested to determine if PD-0332991 can inhibit the proliferation of BSG tumor cells *in vivo*. We treated two cohorts of Ink4a-ARF deficient BSG-bearing mice with PD-0332991 or vehicle (n= 6 and n=7, respectively) for 48 hours (2 daily doses) and then measured the proliferation of BSG tumor cells 24 hours after the second dose by quantifying the percent of phosphoH3 (pH3)-positive cells in the tumors, a marker for M phase (Figure 3B). Here we noted that PD significantly inhibited the proliferation of BSG cells (p=0.0047; Figure 3C). We also investigated whether there was target inhibition *in vivo* after two doses of PD at 4 hours and 24 hours post final dose but we did not observe a correlation between pRb inhibition and cell cycle arrest (data not shown). In addition, we immunostained PD-treated and vehicle-treated tumor sections for cleaved caspase 3, a marker for apoptosis, and we did not observe a significant change in cleaved caspase 3 levels between the two cohorts. In summary, PD is primarily a cytostatic drug *in vivo* and *in vitro* in the Ink4-ARF deficient BSG model.

### PD significantly prolongs survival in the Ink4a-ARF deficient BSG model

**Figure 4:**
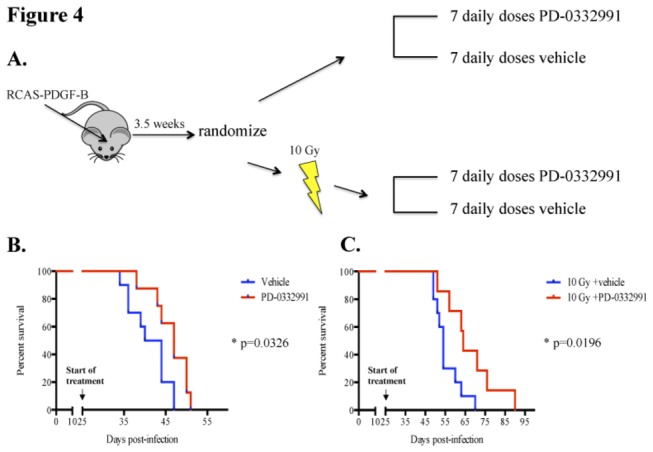
Treatment with PD-0332991 significantly prolongs survival in the Ink4a-ARF deficient BSG model. (A) A schematic of survival study treatment plan. Postnatal day 3-5 pups were infected with RCAS-PDGF-B. Mice were randomized at 3.5 weeks post-infection and either treated with PD (150mg/kg) or sodium lactate, once daily for 7 days, or given one dose of 10 Gy irradiation then the following day started treatment with 7 daily doses of drug or vehicle. Mice were monitored daily and sacrificed when mice were moribund and/or lost 25% body weight. (B) Drug alone prolonged survival by 5 days in comparison to vehicle treated mice (p=0.03 by log-rank). (C) Survival benefit was 10 days (p=0.02 by log-rank) when PD treatment was initiated 24 hours post one dose of 10 Gy.

Next, we were interested to determine if PD can significantly prolong the survival of BSG-bearing mice as a single agent or in combination with a single dose of 10 Gy RT. RCAS-PDGF-B infected mice were randomized to treatment with PD-0332991 or vehicle for 7 days and then monitored for symptoms of tumor formation (Figure 4A). We also treated RCAS-PDGF-B infected mice with a single dose of 10 Gy and then randomized to a 7-day treatment with PD-0332991 or vehicle to begin 24 hours post RT (Figure 4A). Mice in this model have discernible tumors by 4 weeks post induction based on MRI studies previously reported [9], and they develop rapidly-progressive symptoms due to the brain tumor between 4 and 6 weeks post induction. We therefore chose a short 7-day treatment course starting at 3.5 weeks post induction in order to ensure that mice in all groups would complete their respective courses.

The median survival for the vehicle alone group was 42 days vs. 47 days for the PD-0332991 alone group (p=0.033 by log-rank test; Figure 4B). Interestingly, the median survival of the RT + vehicle group was 54 days vs. 64 days for the RT + PD-0332991 group (p=0.02 by log-rank test; Figure 4C). Thus, a 7-day course of PD-0332991 significantly prolongs survival by 12% and a 7-day course of PD-0332991 24 hours post a single dose of 10 Gy RT significantly prolonged survival by 19%.

## Discussion

PD-0332991 is one of several CDK4/6 inhibitors currently in clinical development for the treatment of cancer. Early phase I and phase II studies suggest that this drug is well tolerated and demonstrates efficacy in cancers that are driven by CDK4 and CDK6 such as mantle cell lymphoma [13]. Most importantly, clinical experiences with PD thus far in phase I/II studies for adults with cancer have demonstrated a good safety profile [11]. At the moment, there are no open pediatric clinical trials with this agent for pediatric high-grade gliomas and in particular DIPG. Therefore, the rationale for this study was to use our genetically engineered mouse modeling system to determine the efficacy of PD in pediatric BSGs.

Here we noted that both of our BSG mouse models overexpress CDK4/6 and all three D-type cyclins. As PD can inhibit CDK4/cyclinD1, CDK4/cyclinD3, and CDK6/cyclinD2 at low nanomolar concentrations, we were intrigued to determine if PD would be effective in our BSG models. Our results demonstrate that PD-0332991 is significantly more efficacious at inhibiting cell growth of PDGF-B; Ink4a-ARF deficient BSG cells through inhibition of pRb. *In vitro* cell cycle analysis suggests this is due to a cytostatic effect with cells halting in G0/G1, which is consistent with *in vivo* immunohistochemistry for phospho-H3 and cleaved caspase 3. However, in *vitro* assays of caspase 3/7 activities indicate a very small but significant increase in apoptosis at 2µM and 5µM, which we attribute to the high sensitivity of the assay.

Our data indicate that PD is more efficacious against Ink4a-ARF deficient BSG cells than p53 deficient cells. Importantly, Rb phosphorylation of serine 780, a CDK4/6 phosphorylation site, was inhibited by PD only in the Ink4a-ARF deficient cells but not in the p53 deficient cells. The exact mechanism for the differential response is not clear. Previous studies have shown that hyperphosphorylation of Rb requires both cyclin complexes D1-CDK4/6 and E-CDK2 [14]. Both cyclin D1-CDK4/6 and cyclin E-CDK 2 are capable of phosphorylating Rb, however neither is sufficient to hyperphosphorylate and in turn inactivate Rb. The process of Rb hyperphosphorylation first requires partial phosphorylation by cyclin D1-CDK4/6. This then enables phosphorylation by cyclin E-CDK2 and inactivation of Rb. As Ink4a is an endogenous inhibitor of CDK4/6 and because it is absent in PDGF-B; Ink4a-ARF deficient cells, it is likely that these cells do require CDK4/6 as described above to proliferate and thus PD induces cell cycle arrest. On the contrary, PDGF-B; p53 deficient cells already harbor an endogenous CDK4/6 inhibitor, Ink4a, and therefore it is likely these cells have already found a way to circumvent the requirement for CDK4/6 to proliferate, and as a result PD is less effective. In summary, our observations suggest that Ink4a-ARF deficient BSG cells require CDK4/6 to cycle while p53 deficient cells do not.

*In vivo* testing with PD in PDGF-B; Ink4a-ARF^-/-^ tumor bearing mice caused a significant cell cycle arrest after only two doses. Surprisingly, we did not observe a correlation between pRb and cell cycle arrest *in vivo*. This suggests that cell cycle arrest may be induced by some other mechanism *in vivo* as CDK4/6 is known to phosphorylate other target proteins besides Rb such as FoxM1 [15], or more likely, a change in pRb levels is best detected sometime between 4 hours and 24 hours. Since short-term treatment with PD provided evidence that PD can successfully reach the tumor in the brainstem and inhibit cell growth we were interested to determine if PD would prolong the survival benefit of PDGF-B driven, Ink4a-ARF deficient BSGs. Our results demonstrate a significant increase in survival of PD-treated versus vehicle-treated mice. Furthermore, the survival benefit with PD was also present when PD was administered in conjunction with RT. Our results demonstrate the first therapeutic regimen that provides an improved survival benefit relative to RT alone in preclinical trials for BSG.

As our BSG model is a pediatric model, our results suggest that PD-0332991 should be evaluated in clinical trials for children with Ink4a-ARF deficient gliomas including DIPGs. While Ink4a-ARF deletion at the genomic level is rare in DIPGs [4-7], there is evidence that Ink4a protein expression is lost through other mechanisms [6,8]. DIPGs that lose Ink4a protein expression through other mechanisms may also respond well to PD. In addition, as secondary gliomas in children have a high frequency of Ink4a-ARF loss and PDGFRα amplifications, PD-0332991 may be particularly suitable for this population [16].

Our results are consistent with recent publications in several preclinical glioma models and other tumor types such as malignant rhabdoid tumors noting that Ink4a-ARF loss is a biomarker for response to PD-0332991 [17-21]. Interestingly, potential synergy between PD-0332991 and RT has been noted in adult glioma xenograft studies as well, although in those studies PD-0332991 was either given concurrently with RT or prior to RT [20].

In summary, our results in genetically engineered mouse models of BSG suggest that PD-0332991 may be efficacious in the treatment of pediatric gliomas that have Ink4a-ARF loss. While the PDGF-B; p53 deficient BSG model was relatively resistant to PD, evaluating PD in both models allowed us to recognize that Ink4a-ARF loss is a biomarker for therapeutic response to PD. Our results support the notion that DIPG is a heterogeneous disease and a biopsy at diagnosis is necessary to guide which therapeutic agents will be most efficacious. By evaluating DIPG biopsy tissue for Rb and Ink4a protein expression, patients that are most likely to benefit from PD can be identified (those that express Rb and do not express Ink4a). It is worth noting that there may be other biomarkers for response such as amplification of D-type cyclins, CDK4 or CDK6 amplifications- all of which are present in DIPGs and other pediatric high-grade gliomas [3–5,16,18,20]. Lastly, with the recent identification of histone mutations in pediatric gliomas and particularly in DIPGs, future studies from our laboratory will determine how histone mutations influence response to PD-0332991 [22].

## Materials and Methods

### Mice

Nestin tv-a (Ntv-a) and Ntv-a;Ink4a-ARF^*−/−*^ mice have been previously described by Becher OJ et al. Ntv-a; p53^fl/fl^ mice were created by crossing Ntv-a mice with p53^fl/fl^ (C57BL/6J background) from Jackson Labs.

### Generation of BSGs

To generate Ink4a-ARF^−/−^ BSGs, Ntv-a;Ink4a-ARF^*−/−*^ mice were injected with 1 µL (10^5^ cells) of RCAS-PDGF-B–expressing DF1 cells (described below). To generate p53-deficient BSGs, Ntv-a; p53^fl/fl^ mice were injected with 1 µL of a 1:1 cocktail of RCAS-PDGF-B and RCAS-Cre expressing cells. Injections were made 2mm posterior to the bregma along the midline using a Hamilton syringe and custom needle. Injections were performed on postnatal day 2 – postnatal day 5 mice after being anesthetized on ice. Mice were carefully monitored for the appearance of tumor symptoms (enlarged head, ataxia, weight loss). Upon the appearance of brain tumor symptoms and/or 25% weight loss, mice were euthanized with CO_2_, brains were extracted and either processed for cell culture (described below), snap frozen, or fixed in 10% formalin and paraffin embedded.

### Cell Culture

DF1 cells were purchased from ATCC and cultured in DMEM (ATCC) supplemented with 10% FBS, 2mM L-glutamine, 100 units/mL penicillin and 100 µg/mL streptomycin, and incubated at 39°C and 5% CO_2_. Cells were transfected with RCAS plasmids (RCAS-PDGF-B, RCAS-Cre) using Fugene 6 or X-TremeGENE 9 (Roche) per the manufacturer’s instructions. Cells were used for injections after being passaged at least 6 times from the time of transfection.

To generate BSG cell lines, tumors were isolated from symptomatic mice (described above), enzymatically digested in Earl’s Balanced salt solution containing 4.7mg papain (Worthington) and 60 µg/mL DNAse (Sigma Aldrich). Digestion was inactivated with ovomucoid (0.7mg/mL) (Worthington) containing 14 µg/mL DNAse. Cells were consecutively washed, triturated, and strained to obtain a single cell suspension. The cells were cultured in DMEM supplemented with 2mM L-glutamine, 100 units/mL penicillin and 100 µg/mL streptomycin, and incubated at 37°C and 5% CO_2_.

### Statistical Analysis

All statistical analysis was performed with Graph Pad Prism 4 and 6 software.

### Cell viability

Cell viability was assessed by an MTT assay. Cells were plated in quadruplicate in a 96-well plate (4x10^3^ cells/well in 100 µL) and allowed to adhere for 24 hours. Cells were then treated with PD-0332991 (Pfizer and Selleck) or 0.1% DMSO for 48 hours. Subsequently, an MTT assay was performed as first described by Mosmann [23]. Absorbance was read using Molecular Devices Versa Max Tunable Microplate reader at 540nM.

### Cell Proliferation

A bromodeoxyuridine (BrdU) based cell proliferation ELISA assay kit (Roche) was used to look at proliferation. The cells were plated and treated as described for cell viability assays and then processed according to the manufacturer’s protocol. Absorbance was read using Molecular Devices Versa Max Tunable Microplate reader at 370 and 492nM.

### Cell Cycle Analysis

Cell cycle distribution was analyzed using flow cytometry. Cells were treated with PD-0332991 or DMSO for 48 hours then harvested and washed with phosphate-buffered saline (PBS). Subsequently, cells were fixed with ice-cold 70% ethanol/0.05% Triton X-100/PBS and placed in -20°C overnight. Cells were washed with PBS and resuspended in 0.05% Triton/PBS containing 50 µg/mL RNase A and 20 µg/mL propidium iodide. Cells were then incubated in the dark at room temperature for one hour then sent to the Duke University Cancer Center Flow Core for flow cytometry. A Becton Dickinson FACSCalibur machine was used and results were analyzed using their CellQuest software.

#### Apoptosis Assay

Cells were plated in quadruplicate in a white-walled, clear bottom 96 well plate (4x10^3^ cells/well in 200 µL) and allowed to adhere for 24 hours. The current media was removed and cells were then treated with 100 µL of media containing PD-0332991, 0.1% DMSO (vehicle) or Dasatinib (positive control). Cells were then incubated at 37°C and 5% CO_2_ for 16 hours. Apoptosis assays were conducted using Promega ApoTox-Glo Triplex Assay (# G6321) according to the manufacturer’s instructions, and a Turner Biosystems Modulus Microplate Reader. Caspase 3/7 activity levels were then normalized to their own viability levels for each well. Normalized apoptosis levels were then made relative to the vehicle (0nM PD). Results are graphed as mean with SEM from 3 independent experiments. Statistical significance was determined using One-way ANOVA; paired student’s t-test was used to compare within groups.

### Western Blotting

Lysates from murine BSG cell lines were prepared using a nuclear lysis buffer containing: 50mM Tris, pH 7.5, 0.5M NaCl, 1% NP-40, 1% DOC, 0.1% SDS, 2mM EDTA, protease inhibitor cocktail and phosphatase inhibitor cocktail 2 (Sigma). Lysates were separated by SDS-PAGE then transferred to a nitrocellulose membrane using Invitrogen iBlot. Membranes were blocked in 5% non-fat milk and 0.1% TBS/Tween-20 at room temperature for one hour. Primary antibodies were prepared in 5% BSA/TBS-T and incubated overnight at 4°C. Secondary antibodies conjugated with horseradish peroxidase were prepared in blocking buffer and incubated at room temperature for one hour. Detection was performed with Amersham ECL Plus Detection kit according to the manufacturer’s protocol (GE Healthcare). The following antibodies used were purchased from Cell Signaling Technology: pRb (#8180), Cyclin D1 (#2978), cyclin D2 (#3741), cyclin D3 (#4129), CDK4 (#2906), CDK6 (#3136). Actin (sc-1616) and α-Tubulin (#T6074) were purchased from Santa Cruz and Sigma, respectively.

### In vivo Experiments

This study was carried out in strict accordance with the recommendations in the Guide for the Care and Use of Laboratory Animals. The protocol was approved by the Duke University Animal Care and Use Committee (Protocol Number: A239-10-09). All radiation therapy was performed under isoflurane gas anesthesia, and all efforts were made to minimize suffering.

### Short-term Treatments

Symptomatic mice were treated with two doses of PD-0332991 (150mg/kg) (Pfizer and Selleck) or 50mM sodium lactate, pH 4 (Sigma) administered once daily by oral gavage. Mice were sacrificed 24 hours after their final treatment via CO_2,_ their brains extracted, and either snap frozen or formalin fixed and paraffin embedded.

### Immunohistochemistry

Formalin fixed brains were paraffin embedded by Duke Pathology Core Services. Sections were cut 5µm thick using a Leica RM2235 Microtome. Immunohistochemistry was performed using an automated processor (Discovery XT, Ventana Medical Systems, Inc.) Antibodies used: Anti-phospho-Histone H3 (Ser10) (Millipore 06-570) and Cleaved Caspase 3 (Asp175) (Cell Signaling #9661).

### Therapeutic Study

Ntv-a;Ink4a-ARF^−/−^ mice were treated with radiation therapy + drug, radiation therapy + vehicle, drug alone and vehicle alone. The treatment regimen began 3.5 weeks post-infection with RCAS-PDGF-B. Mice that were irradiated received one dose of 10 Gy at 3.5 weeks using a micro-CT/microirradiator (225Cx, Precision X-ray). The system was commissioned as previously described [24]. Mice received two doses of 5 Gy through two parallel, opposed lateral fields after alignment was documented by fluoroscopy. Irradiation was performed with a collimating cone to produce a rectangular radiation field of 4cm x 1.5cm with an average dose rate of 300 cGy/min at target depth with a 225-kVp, 13-mA beam and a 0.3-mm Cu filter. Mice received isoflurane anesthesia during the radiation therapy. The next day mice were treated for 7 days with PD-0332991 or sodium lactate. Mice were then monitored daily and sacrificed upon moribund condition or 25% weight loss.
